# The effect of improvisational music therapy on the treatment of depression: protocol for a randomised controlled trial

**DOI:** 10.1186/1471-244X-8-50

**Published:** 2008-06-28

**Authors:** Jaakko Erkkilä, Christian Gold, Jörg Fachner, Esa Ala-Ruona, Marko Punkanen, Mauno Vanhala

**Affiliations:** 1 University of Jyväskylä, Finnish Centre of Excellence in Interdisciplinary Music Research, Department of Music, P.O Box 35, FI-40014, University of Jyväskylä, Finland; 2 University of Bergen, The Grieg Academy Music Therapy Research Centre, 5020 Bergen, Norway; 3 Central Finland Health Care District, Keskussairaalantie 19, 40620 Jyväskylä, Finland

## Abstract

**Background:**

Music therapy is frequently offered to individuals suffering from depression. Despite the lack of research into the effects of music therapy on this population, anecdotal evidence suggests that the results are rather promising. The aim of this study is to examine whether improvisational, psychodynamically orientated music therapy in an individual setting helps reduce symptoms of depression and improve other health-related outcomes. In particular, attention will be given to mediator agents, such as musical expression and interaction in the sessions, as well as to the explanatory potential of EEG recordings in investigating emotion related music perception of individuals with depression.

**Methods:**

85 adults (18–50 years of age) with depression (ICD-10: F 32 or F33) will be randomly assigned to an experimental or a control condition. All participants will receive standard care, but the experimental group will be offered biweekly sessions of improvisational music therapy over a period of 3 months. A blind assessor will measure outcomes before testing, after 3 months, and after 6 months.

**Discussion:**

This study aims to fill a gap in knowledge as to whether active (improvisational) music therapy applied to people with depression improves their condition. For the first time in this context, the mediating processes, such as changes in musical expression and interaction during the course of therapy, will be objectively investigated, and it is expected that the results will provide new insights into these processes. Furthermore, the findings are expected to reveal whether music related emotional experiences, as measured by EEG, can be utilized in assessing a depressive client's improvement in the therapy. The size and the comprehensiveness of the study are sufficient for generalizing its findings to clinical practice as well as to further music therapy research.

**Trial registration:**

ISRCTN84185937

## Background

Depression is the most frequent mental disorder in Finland, and its meaning as an illness from both an economic and a human suffering point of view, is among the biggest challenges of today's health-care system [1]. Prevalence of depression in Finland is estimated at 5–6,5% of the population (about 200 000 people) [2]. Lifetime prevalence is about 20% of the population. Depression is more prevalent in women (7,8%) than in men (4,1%) [3]. In addition to traditional psychiatric services, specific depression projects have been established across Finland as part of which medical professionals, mainly nurses, have been trained to assess and council people suffering from depression.

In Finnish treatment practice, medication together with psychiatric counselling is the most common combination of depression treatments. Psychotherapy is offered as well, but, according to Honkonen et al, [4] it is not often utilized. For example, of patients who retired due to depression between 1993 and1994, and between 2003 and 2004, only 9% and 11%, respectively, had received psychotherapy. This is striking given the fact that many patients suffering from severe or chronic depression do not benefit from antidepressants [5]. In particular, after the acute phase of depression, a patient should be offered the possibility of receiving cognitive and other psychological treatments [6]. Also, Honkonen et al. [4] point out that both the international and national clinical guidelines recommend that, along with an appropriate medication, the meaning of interactive treatments, such as psychotherapy, is important.

As regards medication, antidepressants such as SSRIs (selective serotonin reuptake inhibitors), MAOI (Monoamine oxidase inhibitors) and the older TCAs (tricyclic antidepressants) are mostly used [7]. However, side effects as nausea, diarrhoea, headache or anxiety are well known, and patients are therefore seeking alternative complementary treatments [8]. Music therapy has the potential to serve as an adjunct to, or facilitator of, medication, may reduce the amount of medication administered, or can even serve as a method of choice instead of medication, as demonstrated, for example, in anaesthesia [9].

That music affects the emotions is a well-known phenomena, and modern neuroscience has revealed the subcortical areas involved in emotion processing and emotional disorders [10,11]. In particular, subcortical limbic and frontal areas of the brain are thought to malfunction in depressive states, or even show morphological changes and biochemical dysfunction [12,13]. Here, music reception and interaction in music therapy seems to be a powerful non-invasive tool to stimulate brain systems and affective regulation involved in depression.

EEG studies have shown frontal alpha traces of depression. General discussion has focused on frontal alpha power asymmetry as an index of potential risk for emotion-related psychopathology, as a moderator and mediator of emotion [14], and as state and trait markers of correlated activity measures, reflecting Davidson's claim [15] that left frontal hypoactivation is a stable marker of trait vulnerability to depression and anxiety disorders [12]. As depression is found to be correlated with a hypoactivation of left brain activity [14] this may be attenuated after music therapy as earlier studies on music listening have shown [11,16-18].

After Bruscia, music therapy is defined as "a systematic process of intervention wherein the therapist helps the client to promote health, using music experiences and the relationships that develop through them..." [19]. It is often perceived as a psychotherapeutic method where musical interaction, in addition to verbal discussion, is used as means of communication and expression. The aim of music therapy is to help people with mental health problems to develop relationships, and address issues they may not be able to by using words alone [20]. Improvisational music therapy is based on active, spontaneous music making, often in a therapist-client dyad. The method typically utilizes both non-verbal and verbal expression, and the analytical (or psychodynamic) school in particular emphasizes the meaning of, and the process of dealing with, emotions and emotional processes as an essential part of the therapeutic process [21-25]. Bruscia [26] describes analytical music therapy as the use of words and symbolic music improvisations as a means of exploring the client's inner life and facilitating growth. The model was originally developed for adults with emotional or interpersonal problems. Different schools of psychotherapy have emerged and continue to develop from Freud's original work so that the title psychodynamic is currently often used instead of analytic – however, the fundamental basis of both of the models remains the same.

Several randomised controlled trials (RCTs) have previously examined the effects of music therapy for people with depression. In a recent Cochrane systematic review [27], five RCTs of adequate methodological quality were identified. Most of them compared music therapy to standard care and suggested that music therapy was accepted by people with depression and were associated with improvements in mood. However, three of the five studies were about elderly people [28-30], and one about adolescents [31]. Only one study [32] focused on people of working age (between 21 and 62 years). Music listening was a central working mode in most of the studies. Active music-making, which is often central in music therapy practice, was a central working mode in only one study [28]. Therefore, improvisational music therapy with depressed adults of working age is clearly under-researched. Furthermore, research in this area has to date suffered from methodological weaknesses such as low test power (i.e. too small sample size), inadequate randomisation procedures (e.g. lack of allocation concealment), lack of blinding, and inadequate statistical analysis and reporting.

A more thorough (mixed-effects) meta-analysis of available studies to date was conducted 2008 by Gold C, Solli H, Krüger V, Lie S in a recently submitted paper entitled 'Dose-response relationship in music therapy for people with serious mental disorders: Systematic review and meta-analysis'. The review found significant and large effects for both psychotic and non-psychotic serious mental disorders, and a strong dose-response relationship. Findings suggested that music therapy helps patients to improve general symptoms, depression and anxiety symptoms, and level of functioning, and provided estimates of the effect per session, which can be used in power calculations for future studies. Overall, the review supported the conclusion that music therapy is an effective treatment for depression, and suggested that future research should, besides improving methodological quality, specifically address the working modes and the underlying theories of music therapy approaches.

The aims of the study will be:

1. To examine if improvisational music therapy in addition to standard care helps reduce levels of depressive symptoms (primary outcome) compared to standard care only.

2. To examine with psychiatric tests if music therapy reduces general symptoms, alexithymia, functioning, and quality of life in these patients compared to standard care.

3. To examine if music therapy diminishes frontal asymmetry in rest EEG (as a proxy/indicator of clinical changes in depression level)

4. Provided that significant effects are found: to examine if clinical change is mediated by observable and measurable changes in music (i.e. improvisations recorded in therapy) and its elements, in specific musical features, and in the interaction between the client and the therapist.

5. Provided that significant effects are found: to examine if these effects are mediated by changes in music perception (as measured in topographic EEG focusing on changes in frontal and limbic responses; additionally as expressed in self-ratings).

## Methods

**Figure 1:**
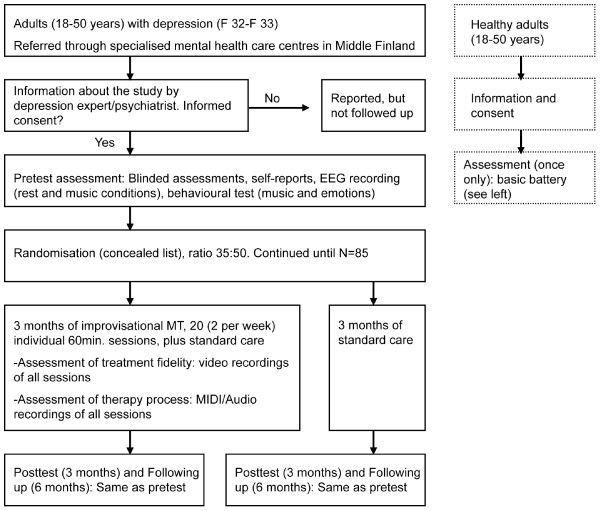
**Overview of the study design**. The design of the study.

This study will be a single-blind randomised controlled trial with two parallel arms. An overview of the study design is shown in Figure 1. The left part of the figure shows how participants eligible for the RCT flow through the study from recruitment to follow-up. In addition, the right part shows the corresponding steps of a separate validation study involving healthy controls. The full methodology of that study is described in a separate article.

### Participants

Participants will be adults (18–50 years of age) with a primary diagnosis of depression (F32 or F33 according to ICD-10), as assessed by Mini-SCID [33] (A structured clinical interview for DSM-III-R) administered by a clinical expert. Participants will be recruited in the Central Finland Health Care District's psychiatric health-centres and the psychiatric policlinics of Jyväskylä city. Participants must be able to complete a questionnaire, and therefore people with insufficient knowledge of the Finnish language or who are otherwise not able to complete such a questionnaire will be excluded.

The ethical board of the Central Finland Health Care District gave their approval on 24^th ^October 2007. Patients will be asked to sign informed consent before they enter the study

### Inclusion criteria

#### Diagnosis F 32 to F33

Participants must have a depression as the primary diagnosis according to ICD F 32 to F33. In this study, depression is the primary focus of interest but because of the frequent comorbidity of depression and anxiety [34,35], also the latter, if occurring with primary diagnosis of depression, is included. Musical skills or any given musical background are not required, although these do not prevent from participation.

### Exclusion criteria

#### a) Repeated suicidal behaviour and psychosis

Patients with a history of repeated suicidal behaviour will not be included because it would presuppose an adjustment of ordinary therapy practice by bringing an additional element with specific concerns with it. The same goes for psychosis.

#### b) Acute and severe substance abuse

Patients who suffer from acute and severe substance abuse will not be included because participation presupposes a commitment for a relatively long time period, as well as an ability to concentrate on various activities included in the study. Addiction problems also add an additional element, which is outside the primary aims of the study.

#### c) Severeness of depression

Patients who are not able to participate in the measurements, nor able to engage in verbal conversation due to severeness of depression, will not be included.

### Interventions

Participants will be randomly assigned to two groups (details in next section). The interventions for both groups will be provided and monitored over the course of three months (2.5 to 3 months) from randomisation.

### Experimental group

#### a) Music therapy

Clients of the experimental group will participate in psychodynamic improvisational music therapy in an individual setting. Music therapy will be conducted twice a week, each session lasting 60 minutes. A total of 20 sessions will be offered, and therapists should aim to complete at least 16 sessions. However, patients with fewer sessions will not be excluded from data analysis (intention-to-treat principle). Active engagement during the therapy process is required for up to 3 months.

The music therapy to be offered is based on the ideas and principles of improvisational music therapy [21,22], and the application of the ideas of Priestley's [36] analytical music therapy. The practical application of the clinical improvisation method has been developed during the training of therapists involved in this research project at JYU Music Therapy Clinic for Research and Training. The basic principle of the intervention is to encourage and engage a client in expressive musical interaction. The starting point for improvisation may be either free or referential. The shared experience is discussed, and the therapeutic process is based on the mutual construction of meaning of emerging thoughts, images, emotional content, and expressive qualities as reflected and understood within the context of the psychodynamic framework.

All improvisations are recorded to computer, and they may be listened to during the same session or afterwards for further processing and discussion. Other than this, no music listening is used as a method; the main focus is on active expressive music making with verbal processing of the experience. Instrumentation is restricted to the use of mallet midi controllers, midi-percussion and modified Djembe-drums (to meet the special needs of data collection in this research project). Every therapy session will be video recorded for data collection and supervision.

The music therapy clinicians who have committed to the study have professional training in music therapy. In addition, they have been specifically trained in the clinical method and it's theoretical basis. This latter training lasted over one year, and was focused on theoretical concepts, the application of clinical improvisation, and process handling issues (building and maintaining a therapeutic relationship, transference & counter-transference, choosing appropriate interventions, verbal processing, gaining insight, etc.).

The aim of the training was to achieve more common understanding about theoretic-clinical questions between therapists, and also to develop the therapeutic expertise needed in the improvisational approach of psychodynamic music therapy. Real-time peer observation and reflective group work was used as a primary training method. All the experiential demonstration sessions during the training were video recorded, and were used as reference material for supervision and further processing of emerging themes.

In addition to the training, adherence to the method and competence in it's application will be monitored and maintained through professional supervision of a psychiatrist and a music therapy supervisor, utilizing the therapists' clinical notes and video- and audio-recorded therapy sessions.

#### b) Standard care

Patients will continue to receive treatment as usual while receiving music therapy. Standard care may consist of medication (antidepressants) and psychiatric counselling. The psychiatrist in charge of the patient's treatment will monitor dose and frequency of treatment they receive before randomisation and after 3 and 6 months.

### Control group

#### Standard care

Patients will receive treatment as usual during the study period. As in the experimental group, standard care may consist of medication (antidepressants) and psychiatric counselling, which will be monitored in the same way as described above for the experimental group.

### Sample size

A power calculation was carried out for the primary outcome (depressive symptoms) and secondary outcomes (general symptoms, functioning). The following assumptions were used in the calculation:

- Number of sessions: The number of sessions is an important predictor of the effect size of music therapy and should therefore be included in a power calculation (Gold C et al, Dose-response relationship in music therapy for people with serious mental disorders: Systematic review and meta-analysis, Unpublished). The target number of sessions in this study is 20. As a conservative estimate, it is assumed that participants will attend at least 15 sessions on average.

- Effect sizes: According to the meta-regression models developed (Gold et al, Dose-response relationship in music therapy for people with serious mental disorders: Systematic review and meta-analysis, Unpublished)the expected effect sizes (standardised mean differences) of 15 sessions of music therapy, compared to standard care, for patients with mental disorders, are as follows: depressive symptoms, .75 (a large effect); functioning, .42 (medium effect).

- Number of subjects to be included: With the funding and time frame available, the feasible number of subjects to be included in the experiment will be 85. Of these, it is estimated that up to 10% may drop out during the study, resulting in a total of 76 cases with available data at posttest.

Based on these assumptions, the power of a t-test was calculated using R package pwr [37]. With the effect sizes derived above, alpha = 0.05, and 31 and 45 complete cases in each of the two groups, the power will be 88% for depressive symptoms and 43% for functioning. (This is a conservative estimate because it does not take into account pretest scores.) As depression will be the primary outcome, the intended sample size will be sufficient for an exploratory trial.

After inclusion in the study and pretest assessment, the participants will be allocated to conditions using a computerised randomisation procedure. This will be done by the external expert that has no direct contact with the patients in order to conceal the allocation from the involved clinicians. An overview of the study design is shown in Figure 1.

### Outcomes

The study will use blind ratings and standardised instruments with demonstrated validity, reliability and sensitivity to change. In addition, objective physical measurements as described below will be used.

### Primary outcome: Depression symptoms

Symptoms of depression will be measured with the Montgomery and Åsberg Depression Rating Scale (MADRS) [38]. The MADRS consists of 10 items, and the total score varies between 0 and 60. High joint reliability (0.76 to 0.95) and sensitivity to change have been shown in several studies. Predictive validity for major depressive disorder has been demonstrated, and cut-off scores have been defined for severe, moderate, and mild forms of depression [39].

### Secondary outcomes of general relevance for the patient

• *Anxiety *will be evaluated by the Hospital Anxiety and Depression Scale (HADS) [40]. The anxiety subscale (HADS-A) of this widely used, valid and reliable questionnaire [41,42] consists of 7 items. Scores can range from 0 to 21, and higher scores indicate more anxiety. The HADS is fast and easy to use, and internal consistency of the Finnish version has been demonstrated [43] for Cronbach's alpha (.83). Consistency normally ranges from .78 to 0.93 for the HADS-A [44].

• *General functioning *will be measured using a blind rating with the GAF (Global assessment of functioning). The GAF is probably the most often used global assessment instrument. Validity in the severely mentally ill population has been demonstrated through high correlation between clinical support and medication levels [45]. The modified GAF, which will be used in this study, has shown high interrater reliability (0.81) [46]. The modified GAF has also been found to be less sensitive to amount of training and variety of employment backgrounds of raters.

• *Quality of life *will be evaluated by the RAND-36. It maps well-being and functioning on eight dimensions. In a Finnish study, discrimination validity of the questions were supported by the results, after which the correlations of the questions with foreign scales were mostly lower than with specially designed scale. Validity and reliability of the Finnish version of the RAND-36 have been found to be adequate (Cronbach's alpha for all the sub-scales not less than .80; for individual sub-scales mostly over .80, never under .70), and are in line with international studies [47].

*Alexithymia *will be evaluated with the TAS-20, which is a self-report questionnaire for the assessment of alexithymia [48]. Adequate internal validity of translated versions of the TAS-20 has been demonstrated (Cronbach's alphas ranging from .70 to .84), as well as high test-retest reliability (values ranging from .83 to .86) [49-52]. Alexithymia is considered in this study because, despite previous suggestions to the contrary, alexithymia seems not to be only a trait related, but a state related disorder as well, typically occurring with depression. In the study by Honkalampi et al, 92% of the patients with major depressive disorder were alexithymic compared with only 4% of the nondepressed patients [53]. In addition, the BDI scores increased or decreased proportionately with the change in TAS-20 score in both groups.

### Secondary outcomes specifically linked to the assumed mechanisms of music therapy

#### Music analysis

All the improvisations created in the therapy sessions will be recorded either as MIDI-data (mallet midi-controllers and midi-percussion) or as digital audio (Djembe drums). For the analysis of MIDI-data, a computational method called MTTB (Music Therapy Toolbox), specifically developed for the analysis of music therapy improvisations in MIDI-format, will be employed. The MTTB-method enables the automatic extraction of various musical features from an improvisation, as well as the examination of certain aspects of the therapist-client interaction. The method has been successfully utilized in the analysis of clinical improvisations by people with mental retardation [54,55]. For the analysis of digital audio data, a computational method called MIR (Music Information Retrieval) will be employed [56]. Like the MTTB, MIR also enables the extraction of specific musical features from the music, and the examination of musical interaction between therapist and client. MIR has not been utilized in a clinical context before.

#### Video analysis

Video recordings will be used as a resource for the interpretation of results, and for studying the therapy process. Masters and post-graduate students will perform qualitative and quantitative content analyses on this data. Focused microanalysis will be used for specific process-related issues, and for studying emotional transitions during the micro- and macro-level processes [57].

### EEG

EEG measurement will be conducted based on the findings of earlier research on depression. Because of it's time-locked correlations, EEG has become the measure of choice for music and brain research; brain imaging techniques that provide distinct spatial information (such as fMRI and PET), for example, lack time and event-related correlations [58,59].

Depression has been found to be correlated with a hypoactivation of left brain activity [14], and this may be improved after treatment [11,16]. Secondly, a more distinct and variable spectral EEG pattern according to personality [60], and specific reactions of theta variables [61] in the treatment and control group, are expected as mediators of change.

EEG research recordings (pretest directly after inclusion/before randomization, post-test 3 months after randomisation) and its corresponding patient data of the clinical groups will be monitored by a clinical neurologist. Analysis of the EEG will focus on topographic distribution of spectral power and percentage of EEG frequencies.

All subjects will listen to two sets of stimuli:

1.) Ordered, but randomly allocated sets of short pieces of instrumental music characterising 5 basic emotions (tenderness, anger, fear, sadness, happiness).

2.) A randomised order of short pieces of instrumental music comprising excerpts of film soundtracks previously rated and categorised according to the emotional dimensions of valence, tension, and energy [Eerola T: **Mapping Musical Features to Perceived Emotions sing Partial Least Squares Regression [Submitted]**. In *The 10th International Conference on Music Perception and Cognition*. Sapporo, Japan: The Center for Research and Development in Higher Education, Hokkaido University, Sapporo, Japan; 2008].

The EEG data will be correlated with the results of the behavioural test for emotional qualities of music (see below), which will contain the same two data sets. This test will be administered after the EEG investigation. To avoid repetition, the excerpts in both sets will be presented in another random order.

### Behavioural test for emotional qualities of music

An investigation of the emotional qualities of music will be carried out (pretest directly after inclusion/before randomization, post-test 3 months after randomization, and follow-up test 6 months after randomization). In these behavioural experiments, participants will listen to two sets of music excerpts and self-rate them based on emotional qualities and characteristics of music. The music excerpts will be 15 seconds long. The first set of excerpts has been chosen based on basic emotions [62-65]. The second set is based on the three-dimensional model [66] of musical emotions. All stimulus examples have been tested and validated with a non-clinical population [Eerola T: **Mapping Musical Features to Perceived Emotions sing Partial Least Squares Regression [Submitted]**. In *The 10th International Conference on Music Perception and Cognition*. Sapporo, Japan: The Center for Research and Development in Higher Education, Hokkaido University, Sapporo, Japan; 2008]. In the experiments, participants will listen to excerpts in a randomized order, and use a computer and specially-written software to rate them.

### Statistical analyses

Results will be analysed on an intention-to-treat basis, using general linear mixed-effects models and effect sizes with 95% confidence intervals. Mediator processes will be examined using structural equation modelling.

## Discussion

The aim of this study is to investigate the effectiveness of improvisational music therapy in the treatment of depression. The few existing properly conducted RCTs in this context suggest that music therapy may be of benefit, but leave many open questions. For instance, little is known about the effect of music therapy in particular client populations, or about the role of a particular music therapy model or technique in the effect. In addition, more research is needed in order to tease out the possible domain specific benefits of music therapy as compared to other forms of therapy. Taking these premises into consideration, we have arrived at certain definitions and methodological solutions. Primarily, we hope to clarify the benefit of music therapy for working age people with depression. We also want to examine whether active music therapy techniques are effective, as could be assumed based on clinical experience. A specific emphasis will be given to mediating agents such as musical expression and interaction within the therapy sessions, and how they reflect and contribute to possible recovery. To clarify that, we will use music-specific, computational analysis methods with particular motivation to find out how useful they are in detecting therapeutically-relevant musical phenomena, and their significance in interpreting the possible outcome. In addition to traditional outcome measures in psychiatry, we will employ an EEG-method, utilising musical stimuli selected on the basis of the latest knowledge concerning the emotional meaning of music.

When planning the principles and instrumentation for the therapy sessions according to the above-mentioned research goals, we have had to assent to some deviations from the traditional clinical practice that may be considered as limitations. To examine the effect of improvisational music therapy, we will not apply any other clinical techniques, such as listening to composed music, in the research therapy sessions. This may, on occasion, prevent spontaneous and intuitive work with mixed therapy techniques. Furthermore, for effective data collection and therapy fidelity reasons, a limited musical instrumentation will be used in the sessions. In some cases, this may restrict one's search for optimal expressive capacity.

Level of medication may be adjusted during a participants' involvement in the trial. This has to be taken into account concerning the comparison of music perception, and when analysing the expressivity of improvised music.

The strengths of this study are undoubtedly its methodological strictness and the work done for treatment fidelity as well as clearness of the clinical technique. Furthermore, a unique combination of outcome measures is utilized, offering the possibility to make profound interpretations if music therapy is found to be effective. The combination of brain imaging and self-rating methods based on musical stimulus, computational music analysis methods, and psychiatric assessment, results in a complementary and many-sided set of measures allowing access to various underlying determinants of a treatment.

If improvisational music therapy turns out to be effective it offers an alternative form of therapy with some unique contents. For those clients who do not benefit from verbal psychotherapy, music therapy – by its nonverbal ways of expressing and interacting – might offer an appropriate option. In addition, evoking and dealing with emotions is often associated with music therapy, which supposedly fits well to the treatment of emotional disorders such as depression. In addition to gaining an insight into the effectiveness of improvisational music therapy, we also aim to test and further develop new kinds of analysis methods for research and clinical purposes. Our study is without doubt an ambitious project, and represents the first time in music therapy research that such a diverse combination of methods has been used to investigate the manifestation of, and recovery from, illness. At its best, in addition to filling gaps in discipline-specific knowledge, we hope that the study will provide useful information regarding the embodiment and treatment aspects of depression in general.

## Competing interests

Esa Ala-Ruona, Jaakko Erkkilä, Christian Gold and Marko Punkanen are clinically trained music therapists.

## Authors' contributions

JE developed the background and design of the study, created the clinical model and therapeutic principles, and drafted the manuscript. CG performed the power calculation, and contributed to the background and design of the study. JF contributed to the background and design of the study. EA-R and MP helped develop the design of the study, and co-created the clinical model and therapeutic principles. MV helped to develop the design of the study. All the contributed to writing the report, read and approved the final manuscript.

## Pre-publication history

The pre-publication history for this paper can be accessed here:


